# STAT6 inhibition stabilizes induced regulatory T cells and enhances their therapeutic potential in inflammatory bowel disease

**DOI:** 10.1007/s12026-025-09686-7

**Published:** 2025-11-07

**Authors:** Rubén D. Arroyo-Olarte, Flaubert A. Pérez-Noriega, María Fernanda Correa-Pérez, Aranza Mejía-Muñoz, Luis I. Terrazas, Sonia Leon-Cabrera

**Affiliations:** 1 Unidad de Biomedicina. Facultad de Estudios Superiores-Iztacala, Universidad Nacional Autónoma de México, Av. De los Barrios 1, Los Reyes Iztacala, Tlalnepantla, Edo. De México 54090 Mexico; 2 Carrera de Médico Cirujano, Facultad de Estudios Superiores Iztacala, Universidad Nacional Autónoma de México, Av. De los Barrios 1, Los Reyes Iztacala, Tlalnepantla, Edo. De México 54090 Mexico; 3 Laboratorio Nacional en Salud, Facultad de Estudios Superiores-Iztacala, Universidad Nacional Autónoma de México, Tlalnepantla, Edo. De México Mexico

**Keywords:** Colitis, STAT6, Tregs, Foxp3, AS1517499

## Abstract

The development and stability of induced regulatory T cells (iTregs) are essential for their immunosuppressive function and therapeutic potential in inflammatory diseases. Although Treg-based immunotherapy offers promise for restoring immune tolerance, clinical application is limited by the instability and reduced potency of iTregs. STAT6 signaling has been implicated in destabilizing Foxp3 expression, a key marker of Treg identity. Here, we investigated the impact of pharmacological STAT6 inhibition on iTreg differentiation, stability, and function both in vitro and in vivo. Naïve CD4⁺ T cells were differentiated into iTregs under standard conditions or expanded with IL-2 in the presence of the STAT6 inhibitor AS1517499 (AS-iTregs). STAT6 inhibition enhanced iTreg stability, maintaining high expression of Foxp3, CD25, PD-1, and CTLA-4 for up to 10 days, even in inflammatory conditions. AS-iTregs also showed increased mRNA levels of Foxp3, IL-10, TGF-β, and PD-1, and reduced IL-6, IL-1β, and DNMT1 expression—suggesting improved functional and epigenetic stability. In the DSS colitis model, adoptive transfer of AS-iTregs alleviated disease severity, preserved mucosal architecture, and increased goblet cell numbers. Histopathological analysis showed reduced epithelial damage and inflammation compared to controls. Importantly, AS-iTregs did not promote tumor growth in a colitis-associated cancer model. Furthermore, in vivo administration of AS1517499 during acute colitis enhanced Treg expansion, activation, and suppressive function. These findings establish STAT6 inhibition as a promising approach to boost iTreg stability and efficacy, advancing the potential of Treg-based therapies for inflammatory disorders.

**Supplementary Information:**

The online version contains supplementary material available at 10.1007/s12026-025-09686-7.

## Introduction

Inflammatory bowel disease (IBD) is a long-lasting and recurring inflammation of the gastrointestinal tract, mainly including Crohn’s disease (CD) and ulcerative colitis (UC). This disorder can be profoundly debilitating and has the potential to cause life-threatening complications [1], like colitis-associated colon cancer (CAC). Current treatment methods for IBD are far from ideal, making it necessary to develop safer and more effective therapeutic strategies. Regulatory T cells (Tregs) are characterized by the transcription factor forkhead box P3 (Foxp3) expression and play a vital role in maintaining tissue homeostasis by regulating immune responses. Tregs can be generated in the thymus from T cell progenitors, such as natural Tregs (nTregs), or from naive T cells, which become induced Tregs (iTregs) in response to various signals like TCR activation, cytokines (IL-2 and TGF-β1), nuclear hormone receptor ligands, and other tissue factors [2, 3]. However, iTregs generated in vitro are less stable than nTregs and tend to transform into effector T cells, leading to ineffective control of tissue inflammation. While nTregs are more effective in suppressing inflammation, they are challenging to collect in sufficient quantities for therapeutic use, and under certain conditions, they can lose Foxp3 expression and produce IL-17 when exposed to pro-inflammatory cytokines like IL-6 in vivo [4]. Therefore, there is a significant need to develop methods for generating iTregs with enhanced stability; optimal expansion likely also requires the addition of an agent that allows “peripheral conversion” of Tregs but limits conventional T-cell proliferation.

Signal transducer and activator of transcription 6 (STAT6), a transcription factor primarily activated by interleukin-4 (IL-4) and interleukin-13 (IL-13), acts as a global repressor of gene expression during T helper cell differentiation and is involved in the repression of Foxp3 transcription [5, 6]. iTregs developed during STAT6 deficiency showed a higher demethylation status for the FOXP3 Treg-specific demethylated region (TSDR), coupled with lower DNA methyltransferase (DNMT1) mRNA expression, suggesting a more stable phenotype [7]. During in vitro iTreg differentiation and expansion, Foxp3 expression typically decreases under iTreg polarizing conditions. STAT6 phosphorylation during long-term iTreg expansion, driven by a concurrent increase in IL-4 levels in the supernatants, can further decrease Foxp3 expression and promote the expansion of the Tact population (CD4^+^CD25^+^Foxp3^−^) [7]. Additionally, iTregs generated in STAT6 deficiency preserved a stable phenotype and high levels of Foxp3 and CD25 for longer, even during inflammatory conditions, than their WT counterparts. The prolonged stability of Foxp3 expression in STAT6^−/−^ iTregs during expansion was observed and correlated with increased expression of PD1 and TGF-β [7]. In addition, targeted TSDR demethylation using CRISPR-TET1 in STAT6-deficient CD4^+^ T cells induces extensive demethylation of the FOXP3-TSDR, resulting in the generation of stable iTregs with enhanced in vitro suppressive capacity [8]. However, significant hurdles remain before the therapeutic potential of STAT6 deficient-iTregs can be fully harnessed. Additional challenges involve the limited survival of ex vivo-expanded Tregs following transfer into in vivo environments and the diminished suppressive functions after adoptive cell transfer.

In this study, we aimed to increase the suppressive activity in vivo and in vitro of iTregs via stabilizing FoxP3 expression through inhibiting STAT6 signaling with AS1517499. This pharmacological inhibition of STAT6 increased the in vitro and in vivo suppressive ability of iTregs, favoring disease prevention and outcome in a murine model of IBD. 

## Methods

### Mice

Eight- to ten-week-old wild-type (WT) Foxp3^EGFP^ knock-in mice (B6.Cg-Foxp3tm2Tch/J) and STAT6^−/−^ Foxp3^EGFP^ mice, which co-express Foxp3 and enhanced GFP under the control of the native FOXP3 promoter, were bred in our animal house and kept in cages according to our institutional guidelines. All experiments were carried out on age- and sex-matched animals. Animal experimentation protocols were approved by the Facultad de Estudios Superiores Iztacala (FES-I) Bioethics Committee for Animal Research.

### In vitro induction of murine iTregs

Naïve CD4⁺ T cells were isolated from mouse splenocytes from wild type (WT) or STAT6 knockout (STAT6^−/−^) mice using the Naïve CD4⁺ T Cell Isolation Kit (Miltenyi Biotec, Bergisch Gladbach, Germany) and activated with plate-bound anti-CD3 and soluble anti-CD28. Cells were then cultured either in standard medium or Treg differentiation medium, consisting of RPMI (Gibco™) supplemented with 10% (v/v) FBS, 1 × non-essential amino acids (Thermo Fisher), 1 mM sodium pyruvate (Thermo Fisher), 50 μM 2-mercaptoethanol (Thermo Fisher), 100 U/mL recombinant mouse IL-2, and 5 ng/mL recombinant human TGF-β1 (PeproTech). After 5 days, cells were analyzed by flow cytometry (FCM), reseeded onto 96-well plates (200,000 cells per well), and incubated for 48 h. Subsequently, cells were treated with either the STAT6 inhibitor AS1517499 (100 nM) (Sigma-Aldrich), vehicle control (0.1% DMSO), rhIL-6 (30 ng/mL), or all trans-retinoic acid (atRA) (10 nM) for an additional 72 h, completing a total of 8 to 10 days in culture.

Following treatment, cells were washed and analyzed by FCM to determine the percentages of CD4⁺Foxp3⁺ (total Tregs) and CD4⁺CD25⁺Foxp3⁺ (activated Tregs) in each experimental group.

### Surface and intracellular staining and flow cytometry

Immunofluorescence labeling was performed by incubating cells with allophycocyanin (APC)-conjugated anti-mouse CD4, BV-711™ anti-mouse CD25, and PE-conjugated anti-mouse PD-1 antibodies (BioLegend, San Diego, CA, USA), all diluted in FACS buffer (Dulbecco’s phosphate-buffered saline [DPBS] supplemented with 1% fetal bovine serum [FBS] and 0.1% sodium azide). Incubation was carried out at 4 °C for 30 min. Foxp3⁺ cells were identified by direct EGFP fluorescence. For intracellular staining of CTLA-4 (PE-Cy7-anti-mouse CD152, BioLegend) and IFN-γ (APC anti-mouse IFN-γ, BioLegend), cells were stimulated as explained in [7] before surface staining and fixation with 4% paraformaldehyde and 0.25% glutaraldehyde. Cells were then extensively washed with DPBS and subsequently permeabilized using DPBS supplemented with 1% FBS and 0.025% saponin. Following immunostaining, cells were washed and resuspended in DPBS. Samples were acquired using an Attune™ NxT Flow Cytometer (Thermo Fisher, Waltham, MA, USA). Data analysis was performed using FlowJo vX.10 software (Tree Star, Covington, KE, USA). A minimum of 10,000 live cells were gated and analyzed. CD4⁺Foxp3⁺ cells were sorted using a FACSAria™ Cell Sorter (BD Biosciences).

### SDS-PAGE and western blot assay

Protein extracts from cells were obtained by the RIPA 1X buffer supplemented with Complete® inhibitors cocktail (Roche Applied Science, Mannheim, Germany). Protein lysates were resolved by SDS-PAGE and electroblotted onto PVDF membranes. The membranes were blocked and probed with primary antibodies to phospho-STAT6 Y641 (Cell Signaling Technology-56554), STAT6 (ABclonal-A19120), and β-actin (BioLegend).

### RNA extraction and quantitative RT-PCR

Total RNA was extracted from iTregs on day 10 of expansion using TRIzol reagent (Invitrogen, USA) following the manufacturer’s instructions. RNA quality (A260/A280 > 1.8) and concentration were assessed using an Epoch™ microplate spectrophotometer (BioTek). Reverse transcription was performed using the RevertAid First Strand cDNA Synthesis Kit (Thermo Fisher, USA) according to the manufacturer’s protocol. Real-time quantitative PCR (qPCR) was conducted using 1 μL of cDNA per reaction, with the SYBR Green Master Mix Kit (Thermo Fisher, USA) in a CFX96™ Real-Time PCR System (Bio-Rad, Hercules, CA, USA). Relative mRNA expression levels were calculated using the 2−ΔCT method, with 18S as the housekeeping gene. The sequences of the oligonucleotides used in this study are listed in [8].

### In vitro suppression assays

Splenocytes from Balb/c mice (Tresp) were isolated, labeled with CellTrace™ Violet (ThermoFisher) for 20 min following the manufacturer’s protocol, and plated in 96-well plates pre-coated with 5 μg/mL anti-mouse CD3 (BioLegend). CD4⁺Foxp3⁺ cells from CTR-iTregs or AS-iTregs (day 10) were sorted using a FACS Aria™ (ThermoFisher) based on EGFP fluorescence. Purified iTregs (> 95% purity) were co-cultured with Tresp at varying Treg: Tresp ratios, maintaining 100,000 cells per well. Cultures were incubated in RPMI complete medium at 37 °C with 5% CO₂ for three days. Cell proliferation was assessed via FCM. Live cells were gated based on 7-AAD exclusion and further sub-gated by CD4 expression. CellTrace™ Violet staining was used to distinguish between non-divided and divided cell populations, and percentages were calculated accordingly. CD4⁺ iTregs were excluded from the analysis by gating out cells lacking CellTrace™ Violet staining.

### Induction and treatment of intestinal colitis

Colitis was induced in wild-type (WT) Foxp3^EGFP^ mice at 8 weeks of age. Mice were randomized and administered intraperitoneal (i.p.) injection of azoxymethane (AOM) (12.5 mg/kg) (Sigma, USA). After 5 days, 2% DSS (molecular weight, MW: 40 000, Alfa Aesar, Canada) was added to drinking water for 7 days. Mice were then left to rest with normal drinking water for 14 days. After that, mice were subjected to one more cycle of DSS administration for 7 days. For cell transplantation, 13 days post-AOM induction, mice were injected intravenously with 100 μl of saline (vehicle group) or received purified WT-iTregs, AS-iTregs, or STAT6^−/−^ iTregs (3 × 10^5^ cells/mouse, purity of the cells > 95%). Mice that received normal distilled water served as controls. Mice were sacrificed on day 40 after the AOM injection. Body weight change and disease activity index (DAI) score were recorded from day 1 to the end of the study. Mice were evaluated twice weekly for body weight, stool consistency, rectal bleeding, and blood in the stool.

### Development and assessment of acute DSS-induced colitis

Acute colitis was induced in 8-week-old wild-type (WT) Foxp3^EGFP^ knock-in mice by administering 4% dextran sodium sulfate (DSS; MW 40,000, Alfa Aesar, Canada) in the drinking water for 7 consecutive days. Mice in the vehicle group received 100 μL of 0.1% DMSO via intraperitoneal (i.p.) injection. For pharmacological intervention, the DSS + AS group received i.p. injections of the STAT6 inhibitor AS1517499 (10 mg/kg; Sigma-Aldrich) on days 1, 3, and 5. Control mice received normal distilled water without DSS. Mice were evaluated daily for body weight, stool consistency, rectal bleeding, and blood in the stool. All mice were sacrificed on day 7 for tissue collection and analysis.

### Induction of colitis-associated cancer (CAC)

Induced Tregs (iTregs) were sorted and injected intravenously (i.v.) into mice treated with AOM and DSS. CAC was induced by administering azoxymethane (AOM) followed by 2% dextran sodium sulfate (DSS) in drinking water for 7 days. Mice were then left to rest with normal drinking water for 14 days. After that, mice were subjected to two more cycles of DSS administration for 7 days, followed by 14 resting days after each cycle. The vehicle group received 100 μl of saline, while WT-iTregs and AS-iTregs groups were injected intravenously with 3 × 10^5^ cells on days 13, 27, and 41. Mice were sacrificed on day 71 after the AOM injection. Mice that received normal distilled water served as controls. Body weight change and disease activity index (DAI) were recorded from day 1 until the end of the study.

### Histopathological analysis

After sacrifice, the colon was removed, weighed, and fixed in 10% phosphate-buffered formalin. Fixed samples were processed using paraffin embedding, sectioning, hematoxylin, and eosin (H&E), and alcian blue as standard procedures. After laboratory processing, slides were scanned in 40x magnification using a whole-slide brightfield scanner (Aperio AT2, Leica Biosystems, Wetzlar, Germany; PreciPoint M8 microscope, Precipoint, Garching bei München, Germany) and displayed in the Aperio ImageScope 12.3 or Precipoint ViewPoint software. Colonic sections were then blindly assessed for immune cell infiltration, epithelial damage, and alterations in mucosal architecture. The grading system described below resulted in a score of 0 (no inflammation) to 12 (severe inflammation) [9]. The fixed colons were stained using Alcian blue to analyze goblet cells (GCs). GCs were quantified using the ImageJ software; the analysis was performed on 10 crypts per animal.

For immunohistochemical analysis, the sections were deparaffinized in xylene and then rehydrated with graded alcohols and processed as reported previously [10]. The sections were incubated overnight at 4 °C with the respective primary antibodies diluted in 1X PBS (anti-Foxp3, 1:100, GeneTex, Irvine, CA, USA; anti-CD8α, 1:200, AB clonal; anti iNOS, 1:200; Cell signaling) and then developed following the conventional technique. The slides were analyzed using an AxioVert.A1 image capture optical microscope (Carl Zeiss Microscopy GmbH, Oberkochen, Germany). Tissue microphotographs were captured using an AxioCam MRc and ZEN lite 2011 software v.1.0.1.0 (Oberkochen, Germany).

### Statistical analysis

Data analysis was performed using a one-way analysis of variance followed by Bonferroni’s multiple comparisons tests using GraphPad Prism 8 (San Diego, CA, USA). In the case of only two-group comparisons, unpaired two-tailed *t*-tests were done. The data are expressed as the mean ± standard error (SEM): *p < 0.05, **p < 0.01, ***p < 0.001, and ****p < 0.0001.

## Results

### STAT6 Inhibition Stabilizes iTregs and Reinforces Their Suppressive Program

**Fig. 1:**
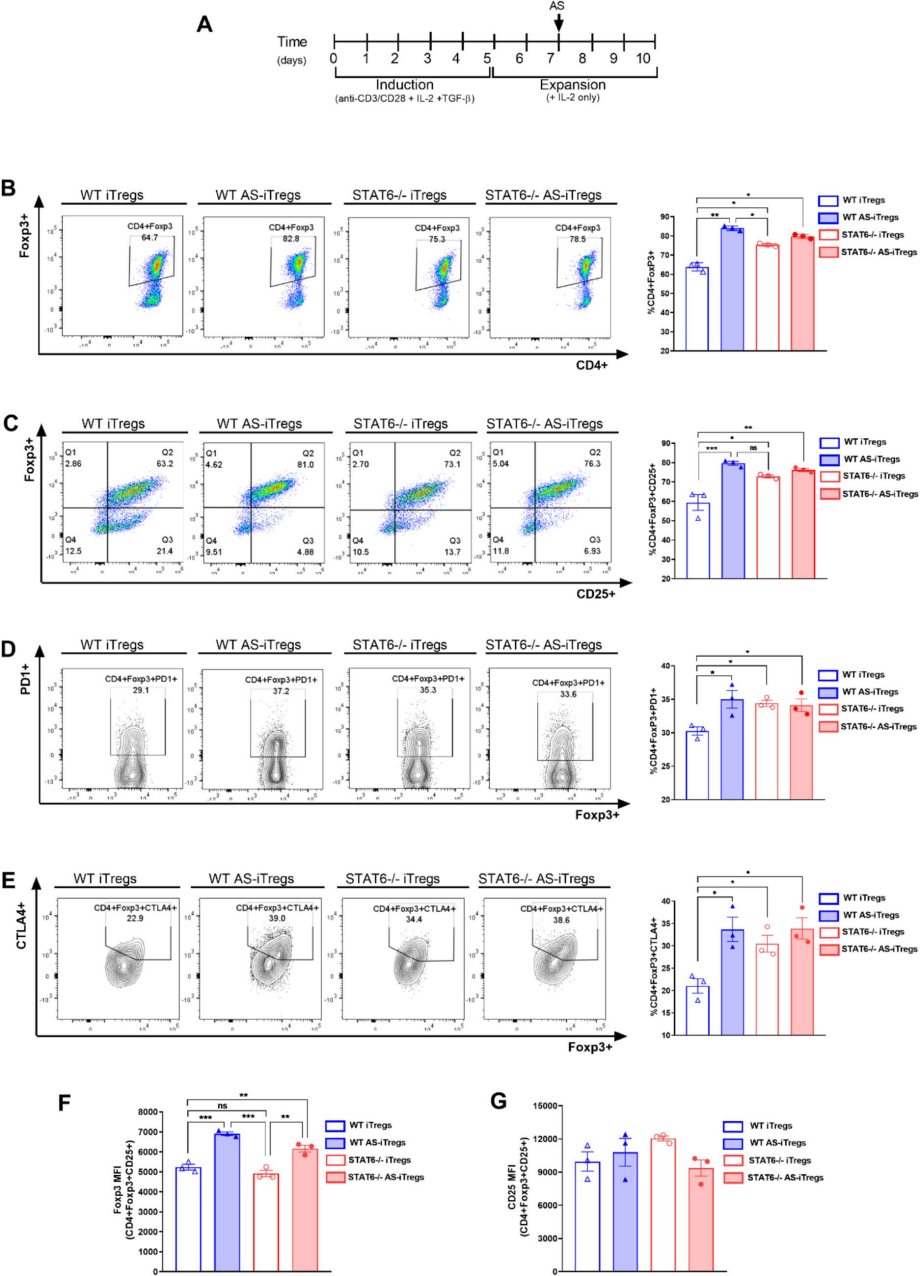
STAT6 inhibition promotes the stabilization of iTreg differentiation in vitro. Naive CD4^+^ T cells were isolated from the spleens of 8–10-week-old WT or STAT6^−/−^ Foxp3^GFP^ mice and differentiated into inducible Tregs (iTregs) over five days (**A**). During this time, the cells were activated with anti-CD3 and anti-CD28 antibodies, TGF-β and IL-2. After five days, TGF-β and the anti-CD3/CD28 antibodies were removed from the culture, and the iTregs were expanded. On day 7, the iTregs were treated with AS1517499 (AS) or left untreated for 72 h. Following this treatment, the cells were harvested and analyzed using flow cytometry (FCM). Representative FCM plots and percentages of CD4^+^Foxp3^+^ cells are shown in (**B**), CD4^+^Foxp3^+^CD25^+^  cells in (**C**), CD4^+^Foxp3^+^PD-1^+^ cells in (**D**), and CD4^+^Foxp3^+^CTLA-4^+^ cells in (**E**). The mean fluorescence intensity (MFI) of Foxp3 (**F**) and CD25 expression (**G**) in WT or STAT6^−/−^ iTregs and those treated with the STAT6 inhibitor (AS-iTregs) are also displayed. *n* = 3 per group; the statistical significance was determined using One-way ANOVA and Bonferroni’s multiple comparison test; *p < 0.05, **p < 0.01, and ***p < 0.001; 4 experimental replicates)

To investigate the effect of STAT6 inhibition on Treg differentiation, we induced iTregs from naive CD4⁺ T cells derived from WT^GFP^ and STAT6^−/−GFP^ mice. The process for T-cell isolation, activation, iTreg induction, and expansion is outlined in Fig. 1A. After a 5-day culture period, the cells were stained with anti-CD4 and anti-CD25 antibodies and analyzed by FCM to evaluate the expression of Treg-specific surface markers. As anticipated, over 85% of the cells successfully differentiated into CD4⁺Foxp3⁺CD25⁺ iTregs (Fig. S1).

To determine whether pharmacological inhibition of STAT6 signaling with AS1517499 promotes iTreg stability in a manner similar to that observed in STAT6^−/−^ mice, we cultured iTregs in the presence of IL-2 and 100 nM of AS1517499 for 72 h (Fig. 1A). To confirm the direct effect of AS1517499 on STAT6 activity in iTregs, we evaluated both total STAT6 and phosphorylated STAT6 (pSTAT6) levels following treatment. Our results demonstrated that 100 nM AS1517499 effectively reduced STAT6 phosphorylation without altering total STAT6 expression (Fig. S2), indicating sufficient inhibition of STAT6 signaling under these conditions. Following ten days of expansion, the frequency of the CD4⁺Foxp3⁺CD25⁺ cells significantly declined to below 59% ± 4 in the WT-iTregs. In contrast, the frequency of iTregs generated with STAT6 pharmacological inhibition (AS-iTregs) remained stable at approximately 80% ± 1.4 after the same period (Fig. 1B, C), with negligible loss in Foxp3 and CD25 expression. As expected, in STAT6^−/−^ animals, the frequency of CD4⁺Foxp3⁺CD25⁺ cells remained consistently high, reaching up to 75% ± 2, and this effect was independent of STAT6 inhibitor treatment (Fig. 1B, C).

The expression of activation and proliferation markers was also accompanied by a marked increase in the co-expression of PD-1 (35.2 ± 2% vs. 30.6 ± 0.5%, *p* < 0.05) and CTLA-4 (33.7 ± 2% vs. 21% ± 2, *p* < 0.05) in WT AS-iTregs compared to WT-iTregs (Fig. 1D, E). Furthermore, WT AS-iTregs showed the highest proportions of Foxp3⁺ and CD25⁺ cells, supporting enhanced Treg stability during in vitro expansion (Fig. 1F, G). Together, these findings indicate that pharmacological inhibition of STAT6 using AS1517499 significantly promotes iTreg differentiation and stability in vitro, recapitulating the phenotype observed in STAT6-deficient mice.

### iTregs induced under STAT6 inhibition remain stable under inflammatory conditions and exhibit enhanced suppression of CD4+ T cells in vitro

**Fig. 2:**
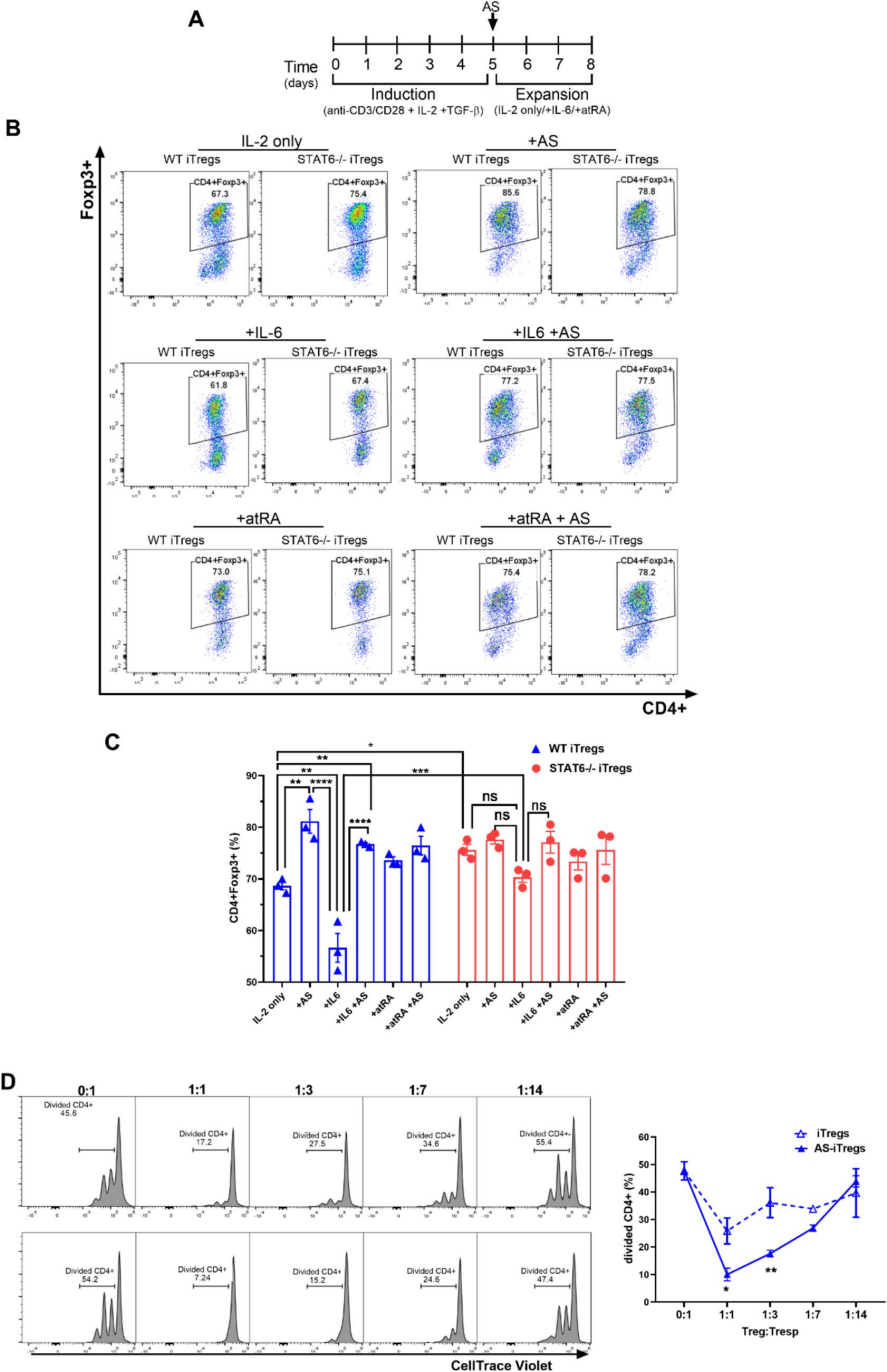
Blocking STAT6 preserves iTreg identity and potentiates suppression despite inflammatory signals. **A** Naive CD4^+^ T cells were isolated from the spleens of 8–10-week-old WT or STAT6^−/−^ Foxp3^GFP^ mice and differentiated into inducible Tregs (iTregs) over five days. During this time, the cells were activated with anti-CD3 and anti-CD28 antibodies, TGF-β and IL-2. After five days, TGF-β and the anti-CD3/CD28 antibodies were removed from the culture, and the iTregs were expanded in the presence of IL-2, AS1517499 (AS), IL-6, or all-trans retinoic acid (atRA) for 72 h. Following this treatment, the cells were harvested and analyzed using flow cytometry (FCM). **B** Representative FCM plots and **C** percentages of CD4^+^Foxp3^+^ cells. Statistical significance was determined using One-way ANOVA and Bonferroni’s multiple comparison test; *p < 0.05, **p < 0.01, ***p < 0.001, and ****p < 0.0001; a total of 3 experimental replicates were performed. **D** Histograms illustrate the proliferation of CD4^+^ responder T cells (Tresp) in the absence or presence of iTregs. iTregs were in vitro generated under Treg polarization conditions from WT mice or treated with 100 nM AS1517499 for 72 h during expansion (AS-iTregs) and FACS cell-sorted CD4^+^Foxp3^+^ cells. Tresp were treated as described in the materials and methods. Numbers indicate the percentage of proliferating cells when iTregs and Tresp cells are co-cultured at different ratios. Graph showing the percentage of total divided CD4^+^ cells at varying iTreg: Tresp ratios. The lymphocyte population was gated based on forward scatter (FSC) and side scatter (SSC) characteristics and further subgated for CD4 expression. Five thousand events were captured from each subgate. The graph shows the mean ± SEM from two experiments with similar results. Statistical significance was determined using a two-tailed unpaired Student’s *t*-test (*p < 0.05, **p < 0.01, 3 experimental replicates)

To assess whether iTregs generated in the absence or inhibition of STAT6 remain stable under inflammatory conditions, we cultured iTregs with the pro-inflammatory cytokine IL-6 for 3 days during the expansion phase (Fig. 2A). In WT iTregs, the frequency of CD4⁺Foxp3⁺ cells significantly decreased in the presence of IL-6 compared to WT iTregs generated under pharmacological STAT6 inhibition (WT AS-iTregs) (55.9 ± 6% vs. 78.0 ± 0.5%, *p* < 0.001) (Fig. 2 B,C). In contrast, STAT6^−/−^ iTregs showed no significant loss of CD4⁺Foxp3⁺ cells upon IL-6 exposure, with comparable frequencies observed between STAT6^−/−^ iTregs and STAT6^−/−^ AS-iTregs (70.3 ± 4.2% vs. 77.5 ± 1.4.%, *NS*), indicating that STAT6 signaling deficiency supports phenotypic stability of iTregs even under cytokine-induced stress (Fig. 2B, C).

Previous studies have shown that all-trans retinoic acid (atRA), a vitamin A derivative, significantly enhances the phenotypic and functional development of TGF-β–induced iTregs and supports their maintenance [11]. To determine whether combining STAT6 inhibition with atRA further improves iTreg stability and function, we co-administered atRA and the STAT6 inhibitor AS1517499 during iTreg expansion (Fig. 2B). However, no significant increase in the frequency of CD4⁺Foxp3⁺ cells was observed in either WT or STAT6^−/−^ cells treated with the combination, suggesting that atRA and AS1517499 do not exert a synergistic or additive effect.

We observed that pharmacological STAT6 inhibition increased the expression of Foxp3, CD25, PD-1, and CTLA-4 in WT AS-iTregs compared to WT iTregs at 10 days of expansion. Therefore, we decided to evaluate whether iTregs developed under these conditions exhibit a superior suppressive capacity.

To assess the suppressive ability of CD4^+^Foxp3^+^ cells, we sorted these cells from the cultures and tested them against naïve splenocytes that were stimulated with α-CD3. The naïve splenocytes were exposed to different ratios of iTreg cells. Upon stimulation with α-CD3, the total splenocytes exhibited strong proliferation of CD4^+^ T cells, as expected (Fig. 2D). Notably, AS-iTregs demonstrated a significantly enhanced suppressive capacity compared to CTR-iTregs, particularly at higher dilutions (1:3 and 1:7) (Fig. 2D). Collectively, these findings suggest that blocking STAT6 signaling not only stabilizes the iTreg phenotype but also enhances their immunosuppressive function.

### STAT6 inhibition results in higher levels of Foxp3, CTLA-4, PD-1, IL-10, and TGF-β mRNA expression during iTreg expansion

**Fig. 3:**
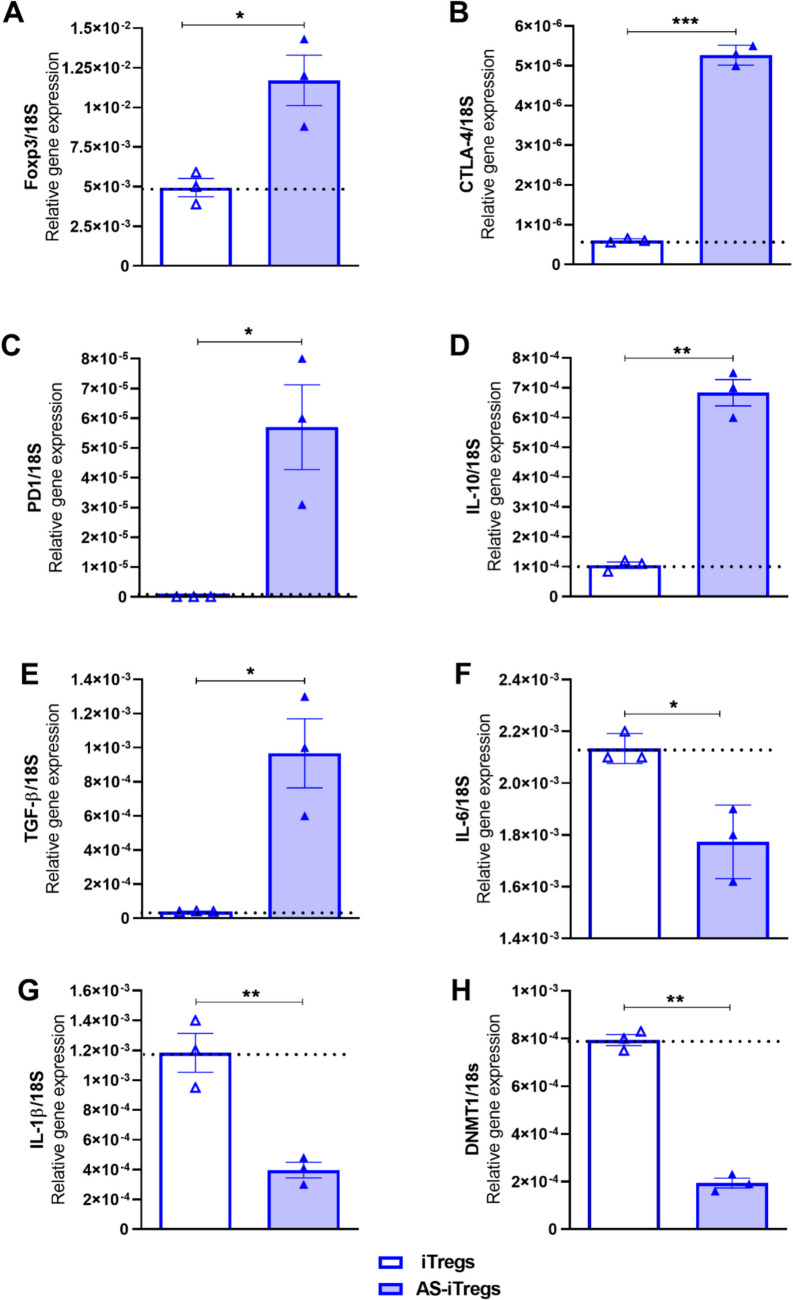
STAT6 modulates the expression of key mRNA programs during iTregs expansion. WT iTregs were expanded as in Fig. 1A and cultured with vehicle or 100 nM AS1517499 (AS-iTregs). At the indicated time point, total RNA was extracted and qRT-PCR performed. The resulting graphs illustrate relative mRNA expression levels for various markers: Foxp3 (**A**), CTLA-4 (**B**), PD-1 (**C**), IL-10 (**D**), TGF-β (**E**), IL-6 (**F**), IL-1β (**G**), and DNMT1 (**H**). Data are expressed as mean ± SEM from two independent experiments that yielded comparable results, assessed using a two-tailed unpaired Student’s *t*-test, with significance indicated as *p < 0.05, **p < 0.01, and ***p < 0.001, 3 experimental replicates

Our findings indicated that iTregs generated from WT mice treated with 100 nM AS1517499 (AS-iTregs) exhibited higher levels of Foxp3 and CD25 during expansion on day 10 of culture compared to control iTregs. Therefore, we further evaluated the mRNA expression of genes considered hallmarks of Tregs. AS-iTregs showed increased relative mRNA expression of Foxp3 and CTLA-4 than CTR iTregs. Both Foxp3 and CTLA-4 are crucial for the function of Tregs in vivo (Fig. 3A, B). A significant elevation in PD-1 mRNA was also observed in AS-iTregs in contrast to control iTregs (Fig. 3C). Notably, AS-iTregs demonstrated a substantial increase, reaching 7–9 times higher levels of CTLA-4 and PD-1 markers compared to iTregs.

We assessed the mRNA expression levels of the cytokines IL-10 and TGF-β, finding that AS-iTregs exhibited a notable increase in the mRNA levels of IL-10 (Fig. 3D) and TGF-β (Fig. 3E) compared to regular iTregs. It is important to note that IL-6 and IL-1β are cytokines that help regulate the balance between Tregs and Th17 cells. IL-6 inhibits TGF-β-induced Tregs differentiation and can downregulate Foxp3, while IL-1β plays a critical role in the early differentiation of Th17 cells [4, 12]. Interestingly, the iTregs expanded with STAT6 inhibitor showed more than a 50% reduction in IL-6 and IL-1β expression levels compared to iTregs (Fig. 3F, G).

TGF-β promotes Foxp3 expression by inhibiting the activity of DNA methyltransferase 1 (DNMT1), which is responsible for maintaining DNA methylation patterns, including those in the regulatory regions of the Foxp3 gene [13]. We also observed that STAT6 inhibition led to a significant reduction in DNMT1 expression in AS-iTregs compared to control iTregs (Fig. 3H). Removing TGF-β from the culture typically results in iTreg instability and increased DNMT1 expression. However, the reduced DNMT1 expression observed in AS-iTregs after 10 days of culture, even in the absence of TGF-β, may contribute to the enhanced stability of Foxp3 expression.

### STAT6 inhibition is crucial for maintaining potent Treg function to suppress colitis

**Fig. 4:**
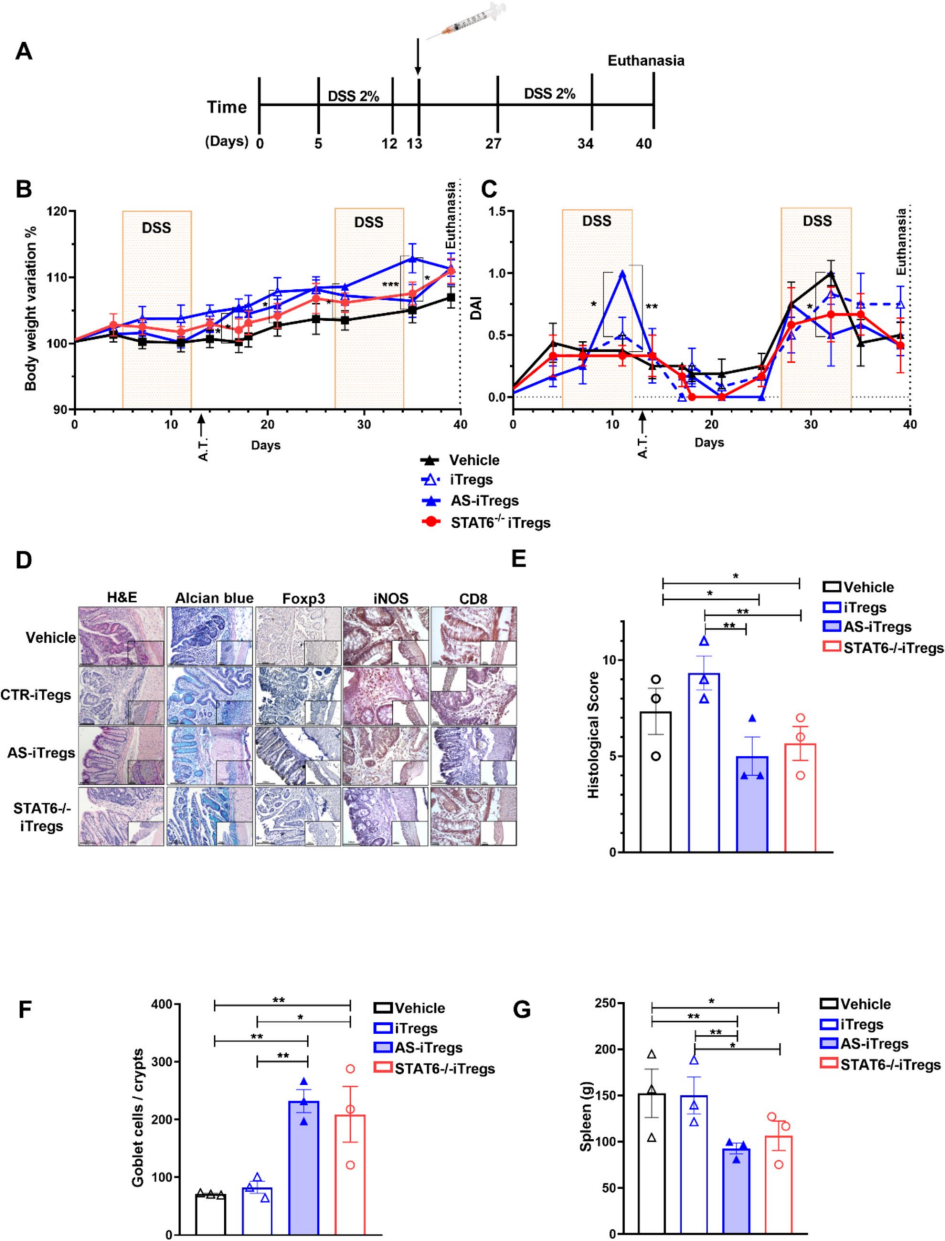
Attenuation of colonic inflammation by adoptive transfer of AS1517499 (AS)-iTregs. **A** Induced regulatory T cells (iTregs) were sorted and intravenously (i.v.) injected into mice subjected to azoxymethane (AOM)/dextran sodium sulfate (DSS)-induced colitis. Colitis was initiated with a single intraperitoneal injection of AOM followed by administration of 2% DSS in drinking water for 7 days, repeated for one cycle of DSS administration. The vehicle group received 100 μL of saline, while control iTregs, AS-iTregs, and STAT6^−/−^ iTregs groups received 3 × 10^5^ iTregs via i.v. injection on day 13. Mice that received only normal drinking water served as healthy controls. **B** Body weight changes, expressed as a percentage of baseline, and **C** disease activity index (DAI) were monitored twice weekly throughout the experiment. **D** On day 40, colon tissues were harvested and analyzed by hematoxylin and eosin (H&E) staining to assess histopathology and by immunohistochemistry for Foxp3, iNOS, and CD8 expression. **E** Histological scores, **F** goblet cell counts (via Alcian blue staining), and **G** spleen weights were quantified. Data are presented as mean ± SEM (*n* = 3 mice per group), representative of two independent experiments. Statistical analysis was performed using one-way ANOVA with Bonferroni’s post hoc test. *p < 0.05, **p < 0.01, and ***p < 0.001

To assess whether iTregs generated in the absence or inhibition of STAT6 remain stable under in vivo inflammatory conditions, we intravenously transferred FACS-sorted control iTregs, AS-iTregs, or STAT6^−/−^ iTregs (3 × 10^5^ cells) into mice during AOM/DSS-induced colitis (Fig. 4A). Following DSS treatment, mice receiving either CTR iTregs, AS-iTregs, or STAT6^−/−^ iTregs exhibited less weight loss (Fig. 4B) and demonstrated reductions in the overall disease activity index (DAI) score (Fig. 4C).

Hematoxylin and eosin (H&E) staining revealed that vehicle-treated mice subjected to AOM/DSS exhibited severe colonic damage, characterized by destruction of the intestinal epithelial structure, crypt loss, inflammatory cell infiltration, and marked edema (Fig. 4D), which was reflected in significantly elevated pathological scores (Fig. 4E). In contrast, mice treated with AS-iTregs showed substantial improvement in histopathological features, with restoration of crypt architecture and reduced inflammation, comparable to the effects observed with STAT6^−/−^ iTregs. Conversely, mice receiving vehicle alone or control iTregs (iTregs) continued to display pronounced crypt disruption, persistent inflammatory infiltrates, and splenomegaly (Fig. 4E–G).

To assess intestinal barrier integrity, Alcian blue staining was performed to detect acid mucins. The gut tissues of mice in the vehicle and control iTregs groups showed a significant reduction in goblet cell numbers. In comparison, AS-iTregs and STAT6^−/−^ iTregs transfection significantly increased the number of goblet cells (Fig. 4F). These findings indicate that AS-iTregs treatment can effectively rescue AOM/DSS-induced gut damage during chronic colitis.

To assess the immunological impact of these transfection therapies, we analyzed immune cell populations implicated in tissue damage and immune regulation in IBD. Treg infiltration was assessed by immunohistochemistry for Foxp3⁺ cells. Only sparse Foxp3⁺ cells were observed in the colonic tissue of mice receiving vehicle, control iTregs, or STAT6^−/−^ iTregs. In contrast, AS-iTreg-treated mice showed a marked increase in Foxp3⁺ Treg infiltration in the colon (Fig. 4D), indicating enhanced local Treg recruitment and potential immunoregulatory activity.

Given that CD8⁺ effector T cells play a key role in epithelial injury during IBD, we also assessed CD8⁺ cell infiltration in colonic tissues. Notably, AS-iTreg therapy was associated with a reduction in CD8⁺ T cell accumulation, whereas substantial CD8⁺ cell infiltration was observed in the colons of mice treated with vehicle, control iTregs, or STAT6^−/−^ iTregs (Fig. 4D).

Additionally, we examined inducible nitric oxide synthase (iNOS) expression, a pro-inflammatory enzyme upregulated during colitis, particularly in inflamed epithelium and infiltrating immune cells. As expected, iNOS⁺ cells were markedly increased in the colons of vehicle-treated mice. Interestingly, no reduction in iNOS⁺ cell numbers was observed in mice treated with control iTregs or AS-iTregs. A decrease in iNOS expression was only evident in the STAT6^−/−^ iTregs group (Fig. 4D). It is important to note, however, that while iNOS upregulation is a hallmark of inflammation in colitis, its role in disease pathogenesis is complex and may vary depending on the stage and subtype of the disease.

### AS-iTregs suppress inflammatory responses without exacerbating tumor development in CAC

**Fig. 5:**
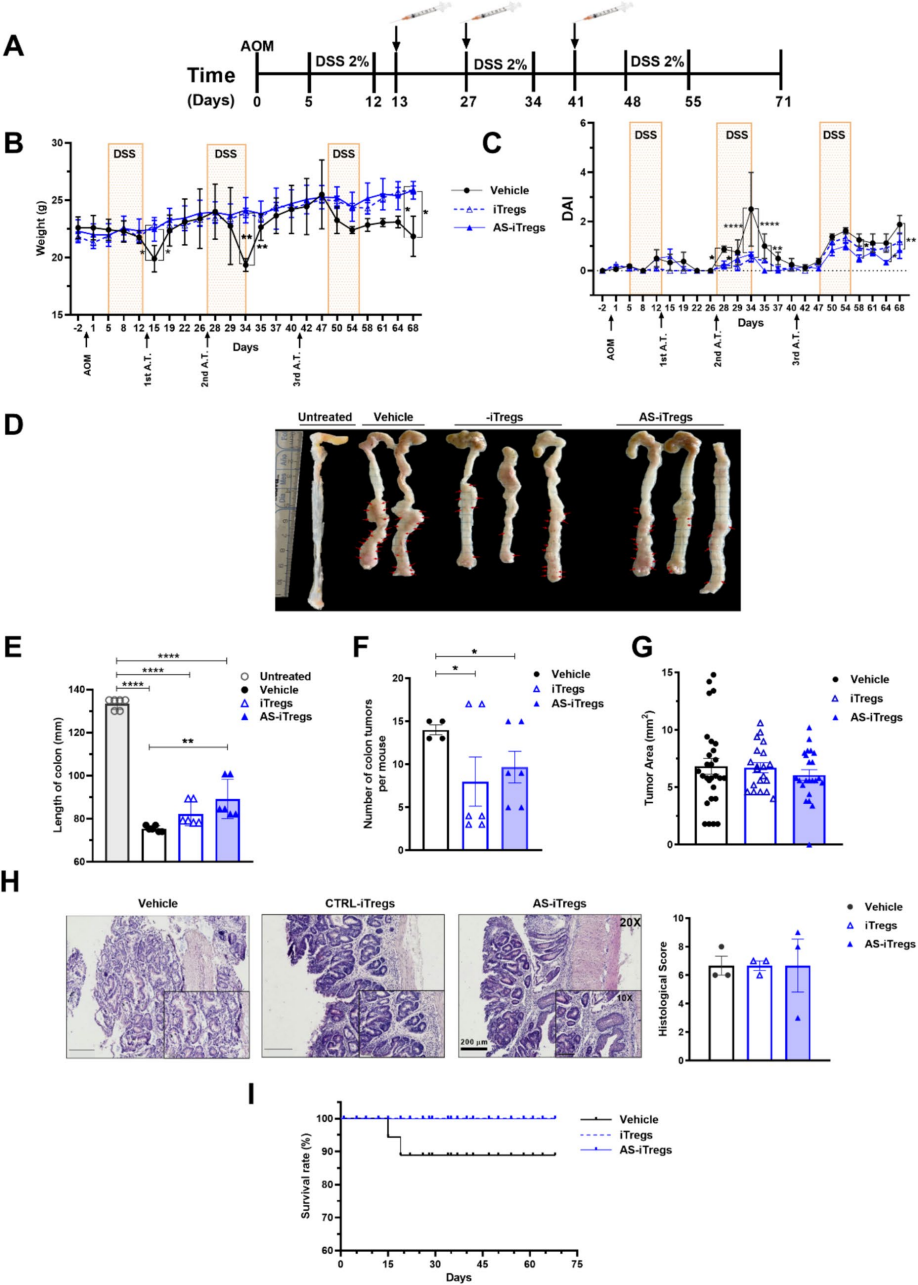
AS-iTregs alleviate inflammation without affecting tumor progression in a CAC model. **A** iTregs were sorted and injected intravenously (i.v.) into mice subjected to AOM/DSS-induced CAC. The vehicle group received 100 μL of saline, while the control iTregs and AS-iTregs groups were injected i.v. with 3 × 10^5^ FACS-sorted cells on days 13, 27, and 41. Mice given normal distilled water served as controls. Effects of iTreg transfer on disease progression: **B** changes in body weight; **C** disease activity index; **D, E** colon length; **F** number of tumors; **G** tumor area; and **H** histological sections of inflamed colons at day 71. **I** Survival rates were recorded daily following CAC initiation. Data are presented as mean ± SEM for 6 mice per group. Similar results were observed in two independent experiments. One-way ANOVA and Bonferroni’s multiple comparison tests (*p < 0.05, **p < 0.01, ***p < 0.001, and ****p < 0.0001)

PD-1 and CTLA-4 can have context-dependent roles in immune regulation, potentially contributing to both immune suppression and tumor progression. To assess whether STAT6 inhibition-induced iTregs (AS-iTregs) could exert excessive immunosuppression or promote tumor growth, we compared the effects of adoptively transferred control iTregs and AS-iTregs in the AOM/DSS-induced colitis-associated cancer (CAC) model (Fig. 5A). iTregs were administered intravenously in three adoptive transfers during the inflammatory phases of CAC development. Mice were sacrificed on day 71, a time point at which adenocarcinomas were well established. This experimental design allowed us to assess the potential impact of AS-iTregs on tumor progression under chronic inflammatory conditions.

AOM/DSS treatment in the vehicle group resulted in significant body weight loss and increased DAI scores. In contrast, mice treated with iTregs or AS-iTregs exhibited a protective effect, including reduced weight loss and improved diarrhea and intestinal bleeding (Fig. 5B, C).

Inflammation associated with colitis typically shortens the colon. AS-iTregs treatment significantly preserved colon length compared to vehicle-treated animals (Fig. 5D, E). As expected, all mice in the vehicle group developed colon tumors. In contrast, both iTregs and AS-iTregs significantly reduced tumor incidence, though neither group showed an effect on tumor size (Fig. 5 F, G). While the colon tissues of iTregs and AS-iTregs-treated mice displayed reduced inflammation, disrupted crypt architecture was still evident, and no differences were observed in total histological score (Fig. 5H).

Notably, a significant difference in survival was observed between mice with or without iTreg treatment (Fig. 5H). However, no differences were observed between iTregs and AS-iTregs-treated mice. Our results demonstrate that STAT6 inhibition enhances iTreg differentiation and mitigates inflammation, though this immunomodulation alone is insufficient to prevent CAC development, highlighting the complexity of tumor-immune interactions in chronic inflammation.

### Pharmacological STAT6 inhibition promotes Treg-mediated immune regulation in acute colitis

**Fig. 6:**
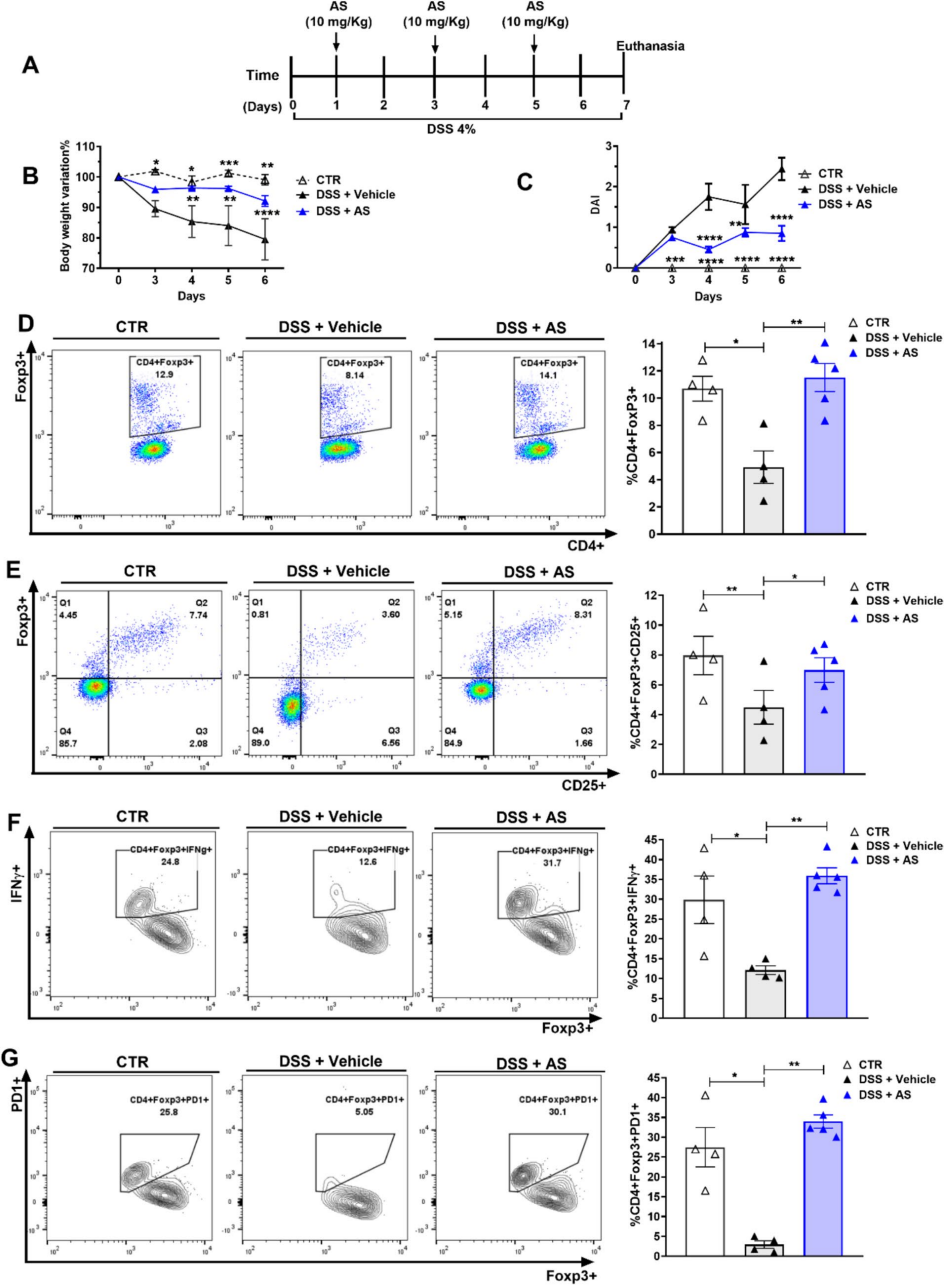
Pharmacological STAT6 inhibition promotes Treg-mediated immune regulation in acute colitis. **A** Acute colitis was induced by administering 4% DSS in drinking water for 7 days. The vehicle group received 100 μL of DMSO, while the AS1517499 (AS) group was treated intraperitoneally with 10 mg/kg of AS on days 1, 3, and 5. A separate group receiving normal drinking water served as healthy controls. **B** Body weight change (expressed as a percentage of baseline) and **C** disease activity index (DAI) were monitored daily. Representative flow cytometry plots and frequencies of **D** CD4⁺Foxp3⁺ cells, **E** CD4⁺Foxp3⁺CD25⁺ cells, **F** IFN-γ⁺, and **G** PD-1⁺ expression in CD4⁺Foxp3⁺ Tregs from mesenteric lymph nodes (MLNs). Data are shown as mean ± SEM (*n* = 4–5 mice per group) and are representative of two independent experiments. Statistical significance was determined using one-way ANOVA followed by Bonferroni’s post hoc test (*p < 0.05, **p < 0.01, ***p < 0.001, and ****p < 0.0001). In panels **B** and **C**, asterisks indicate differences between the DSS + AS group and all other groups

Building on our in vitro findings that STAT6 inhibition enhances the stability and suppressive function of iTregs, we next investigated whether in vivo administration of the STAT6 inhibitor AS1517499 could similarly enhance Treg activity and attenuate inflammation in a model of acute colitis. To this end, we utilized the DSS-induced acute colitis model in WT Foxp3^EGFP^ mice (Fig. 6A). Consistent with previous reports, mice treated with DSS and vehicle exhibited progressive weight loss and a marked increase in DAI over time. In contrast, mice treated i.p. with AS1517499 displayed significantly reduced weight loss and DAI, confirming the anti-inflammatory effects of STAT6 inhibition (Fig. 6B and C), in line with our earlier findings [14].

However, in our previous study, we did not evaluate whether this reduced inflammation was associated with enhanced Treg proliferation or function. Here, we demonstrate that AS1517499 treatment during acute colitis prevents the decline in the frequency of CD4⁺Foxp3⁺ Tregs (Fig. 6D) and promotes the maintenance of activated Treg subsets, including CD4⁺Foxp3⁺CD25⁺ cells (Fig. 6E), IFN-γ–expressing Tregs (CD4⁺Foxp3⁺IFN-γ⁺; Fig. 6 F), and PD-1–expressing Tregs (CD4⁺Foxp3⁺PD-1⁺; Fig. 6G). These results provide novel evidence that pharmacological inhibition of STAT6 in vivo enhances Treg expansion and activation, while supporting their suppressive capacity under inflammatory conditions.

## Discussion

In the present study, we focused on the inducible Tregs (iTregs), addressing whether pharmacological inhibition of STAT6 during expansion could make iTregs stable and therapeutically efficacious in IBD models. We confirmed that the Foxp3 and CD25 expression could be increased by the combination of AS1517499 and IL-2 during iTreg expansion, which led to the expression of PD-1 and CTLA-4, even after 10 days. This finding further highlights the critical role of STAT6 inhibition in regulating iTreg stability.

Previously, we reported in vitro assays using STAT6 knockout mice; iTregs preserved a stable phenotype and expressed high levels of Foxp3 and CD25 during long expansion periods, even in the presence of IL-6 [7]. However, using cells from animals with a permanent deficiency in the STAT6 pathway raises concerns about potential adverse effects on cellular function. In this study, we employ AS1517499, a selective STAT6 inhibitor that targets and prevents the phosphorylation of STAT6. The absence of STAT6 could modify the methylation status of the TSDR. STAT6^−/−^ iTregs have a significantly higher demethylation status of TSDR, which could be related to their enhanced stability and suppressive functions [7]. By inhibiting the phosphorylation of STAT6, AS1517499 effectively blocks downstream signaling events, promoting TSDR demethylation and increasing Foxp3 expression. This makes it a valuable tool for studying STAT6-dependent pathways and a potential therapeutic candidate for inflammatory conditions and other diseases linked to dysregulated Treg immunity.

There is significant variability among Tregs in terms of their development, antigen specificity, activation status, and responses to inflammation [15, 16]. Programmed cell death protein 1 (PD-1) and cytotoxic T-lymphocyte-associated protein 4 (CTLA-4) are expressed by activated T cells, which counteract T cell receptor (TCR) signals and dampen the responses of effector T cells [17]. A subset of effector Tregs also expresses these receptors [18], and elevated levels of PD-1 and CTLA-4 are associated with their suppressive functions [19].

AS-iTregs showed upregulation of PD-1 and CTLA-4 at mRNA and protein levels and downregulation of inflammatory cytokines. PD-1 is dispensable for thymus Tregs development and critical for extrathymic differentiation of peripheral Tregs in vivo [20]. Targeting PD-1 and CTLA-4 does not inherently enhance Tregs’ suppressive activity. Instead, these pathways primarily regulate the expansion of the effector Tregs [18, 21]. These findings suggest that STAT6 inhibition during iTreg expansion enhances the expression of PD-1 and CTLA-4, which play critical roles in the upregulation and stabilization of Foxp3. However, the precise underlying mechanisms require further investigation.

During iTreg development, naïve CD4⁺ T cells receive signals through CD28, IL-2, and TGF-β. These signals induce Foxp3 expression and establish a Treg-specific CpG hypomethylation pattern at key genomic loci, including the TSDR in the Foxp3 gene critical for Treg lineage identity and suppressive function [22]. DNA methyltransferase 1 (DNMT1) plays a pivotal role in regulating DNA methylation patterns at the Foxp3 locus, influencing the development and stability of iTregs. Elevated DNMT1 expression can lead to remethylation of the Foxp3 locus, diminishing Foxp3 expression and causing iTregs instability, particularly under inflammatory conditions or without TGF-β [23]. Our results show that Foxp3 expression in iTregs is strongly induced by TGF-β and IL-2, but its levels decline when TGF-β is removed from culture. Interestingly, adding the STAT6 inhibitor AS1517499 during expansion restores Foxp3 and CD25 levels while reducing DNMT1 expression. These findings suggest that STAT6 signaling indirectly influences DNMT1 expression, as cytokines like IL-4 and IL-13 (which activate STAT6) have been reported to upregulate DNMT1 in other contexts [24]. However, in the context of AS1517499-mediated STAT6 inhibition, the reduction of DNMT1 expression in iTregs is evident. While this study highlights the correlation between STAT6 inhibition and DNMT1 downregulation, the specific mechanisms driving this relationship remain to be fully elucidated.

The pivotal role of STAT6 signaling in Tregs development during colitis and CAC has been highlighted in STAT6 knockout mice, which exhibit increased levels of suppressive Tregs in the early stages of CAC, correlating with reduced tissue damage [7]. However, the potential inhibitory effects of AS1517499 on Tregs differentiation to produce stable iTregs capable of suppressing inflammation in a mouse model of IBD have not been investigated. This study demonstrates that AS-iTregs exhibit significantly greater suppressive capacity than control iTregs in in vitro assays. Furthermore, the adoptive transfer of AS-iTregs into mice with colitis markedly reduced disease-associated parameters. In the colitis model, adoptive cell transfer was performed after the first DSS cycle (day 13), when significant inflammatory responses typically occur in the colon. A single injection of AS-iTregs on day 13 was sufficient to reduce barrier disruption and limit immune cell infiltration into the colon tissue more effectively than regular iTregs. However, we agree that further investigation is warranted to determine whether AS-iTregs retain their therapeutic efficacy during later disease stages, particularly during the chronic phase of colitis following repeated DSS exposure, and future studies employing transfer colitis models in Rag1^−/−^ mice will be crucial to further validate the in vivo suppressive function of AS-iTregs. Exploring this extended therapeutic window would provide important insight into the durability and optimal timing of AS-iTreg-based interventions.

We administered three cell transfers to evaluate the efficacy of AS-iTregs in a CAC model. However, while both AS-iTregs and control iTregs slightly decreased the DAI and tumor count, the iTreg treatment alone was insufficient to halt tumor development completely. While AS-iTregs demonstrate greater suppressive capacity in vitro and express higher levels of suppression-associated markers, their enhanced effectiveness appears limited to controlling inflammation and is insufficient to suppress cancer progression. Importantly, under our experimental conditions, we found no evidence of excessive immunosuppression or enhanced tumor growth associated with AS-iTregs. However, we acknowledge that we did not assess the effects of iTreg transfer during later stages of CAC development, where increased immunosuppression might have pro-tumorigenic effects.

Tregs modulate various immune cell populations, positioning them as a potential therapeutic target for numerous autoimmune diseases, IBDs, and the prevention or treatment of epithelial cancers. However, their role in colon cancer remains highly controversial. While Tregs can regulate local inflammation, the impact of their increased frequency depends on the timing, which can either improve or worsen the patient's prognosis [25, 26]. In the CAC model, the adoptive transfer of iTregs during the advanced stages of the disease could be counterproductive. The overexpression of CTLA-4 by iTregs, which, upon interaction with CD80/CD86 on dendritic cells, could suppress T lymphocyte activation or induce anergy in effector T cells, thereby diminishing anti-tumor immunity. In addition, PD-1 blockade, a standard cancer immunotherapy, can inadvertently amplify PD-1⁺ Tregs, leading to hyperprogressive disease in certain patients [27]. These findings underscore the complex role of PD-1⁺ Tregs in cancer progression. To mitigate these risks, future strategies may involve tailoring iTreg expansion protocols to enhance context-specific function. This could include the generation of gut-homing iTregs with a limited lifespan, engineering iTregs with molecular control switches, or fine-tuning their suppressive potency to allow inflammation resolution without compromising anti-tumor immunity. Such modifications could help achieve a therapeutic balance—controlling pathogenic inflammation in IBD while minimizing the risk of promoting tumor progression in CAC. Our results underscore the need for further research into the functional programming and temporal dynamics of AS-iTregs, with an emphasis on stage-specific effects and long-term outcomes in inflammation-driven tumor models. In this study, we demonstrate that pharmacological inhibition of STAT6 enhances the stability and anti-inflammatory function of iTregs in vivo; however, we did not investigate whether these effects are mediated, at least in part, through metabolic reprogramming. It is plausible that STAT6 inhibition promotes a metabolic phenotype favorable for Treg lineage integrity, such as enhanced fatty acid oxidation or reduced glycolytic flux, as has been observed in other contexts of stable Treg differentiation [28, 29].

Other questions remain to be addressed regarding using STAT6 inhibitors in iTreg development. For instance, supplementing these culture conditions with agents such as DNA methyltransferase inhibitors may enhance the stability of the human Tregs and improve their suppressive capabilities during expansion. By leveraging this approach, antigen-specific Tregs can be developed under STAT6 inhibition, as they retain the same antigen specificity as the activated T effector cells from which they are derived. In the context of IBD, intestinal antigens—including those derived from the microbiota (e.g., flagellins) and inflammation-related molecules (e.g., IL-23R)—are increasingly recognized as relevant targets for Treg-based interventions [30, 31]. While the current manuscript focuses on a strategy to enhance iTreg stability and function through pharmacological STAT6 inhibition during in vitro differentiation, we have not yet evaluated the antigen specificity or off-target effects of AS-iTregs in vivo. Finally, the expansion of Foxp3⁺IFN-γ⁺ Tregs following STAT6 inhibition during acute colitis may reflect an adaptive regulatory phenotype rather than pathogenic plasticity. These cells could contribute to immune suppression in Th1-skewed environments, maintaining Treg function under inflammatory stress. Recently, IFN-γ signaling by Th1-like Tregs has been shown to induce the expression of gut-homing chemokine receptors such as CXCR3 in vivo and CXCL10 in vitro, without losing TSDR hypomethylation state or their suppressive capacity under inflammatory conditions [32, 33].

Our study demonstrates that STAT6 inhibition stabilizes iTregs and enhances their therapeutic potential; however, several limitations remain. We did not directly assess the epigenetic landscape, and future studies employing bisulfite sequencing, ChIP-seq, or ATAC-seq will be needed to define underlying mechanisms. While enhanced suppressive function was confirmed in vitro, additional validation in Rag1^−/−^ transfer colitis models and extension to human iTregs will be essential to establish clinical relevance. Finally, fate-mapping approaches could further clarify the long-term stability and plasticity of AS-iTregs under inflammatory conditions, representing important avenues for future investigation.

## Supplementary Information

Below is the link to the electronic supplementary material.

ESM 1 **Figure S1 Induction and Analysis of iTregs from CD4⁺ Naïve T Cells. A. **Schematic of the 5-day in vitro induction protocol used to generate induced regulatory T cells (iTregs) from CD4⁺ naïve T cells isolated from wild-type (WT) or STAT6⁻/⁻ mice. **B.** Representative flow cytometry (FCM) plots and corresponding bar graphs showing the frequencies of CD4⁺Foxp3⁺CD25+ iTregs on day 5 post-induction. **C.** Representative FCM plots illustrating the gating strategy employed for analysis throughout the study.  (TIF 1.90 MB)

ESM 2 **Figure S2 A.** Representative Western blots showing the protein levels of phosphorylated STAT6 in WT AS-iTregs. The cells were stimulated with 20 ng/ml recombinant murine IL-4 for 20 min and 24 h with and without AS1517499 (AS). B. Densitometry analysis of pSTAT6 expression under the conditions previously mentioned. Densitometry analyses of Western blots show the results of at least two independent experiments. (***p < 0.001, ****p < 0.0001)  (TIF 142 KB)

## Data Availability

No datasets were generated or analysed during the current study.
