# Supplementary figures and images for: STAT6 inhibition stabilizes induced regulatory T cells and enhances their therapeutic potential in inflammatory bowel disease
Source: Immunol Res. 2025 Nov 7;73(1):158. doi: 10.1007/s12026-025-09686-7 (PMC12594747; doi:10.1007/s12026-025-09686-7)

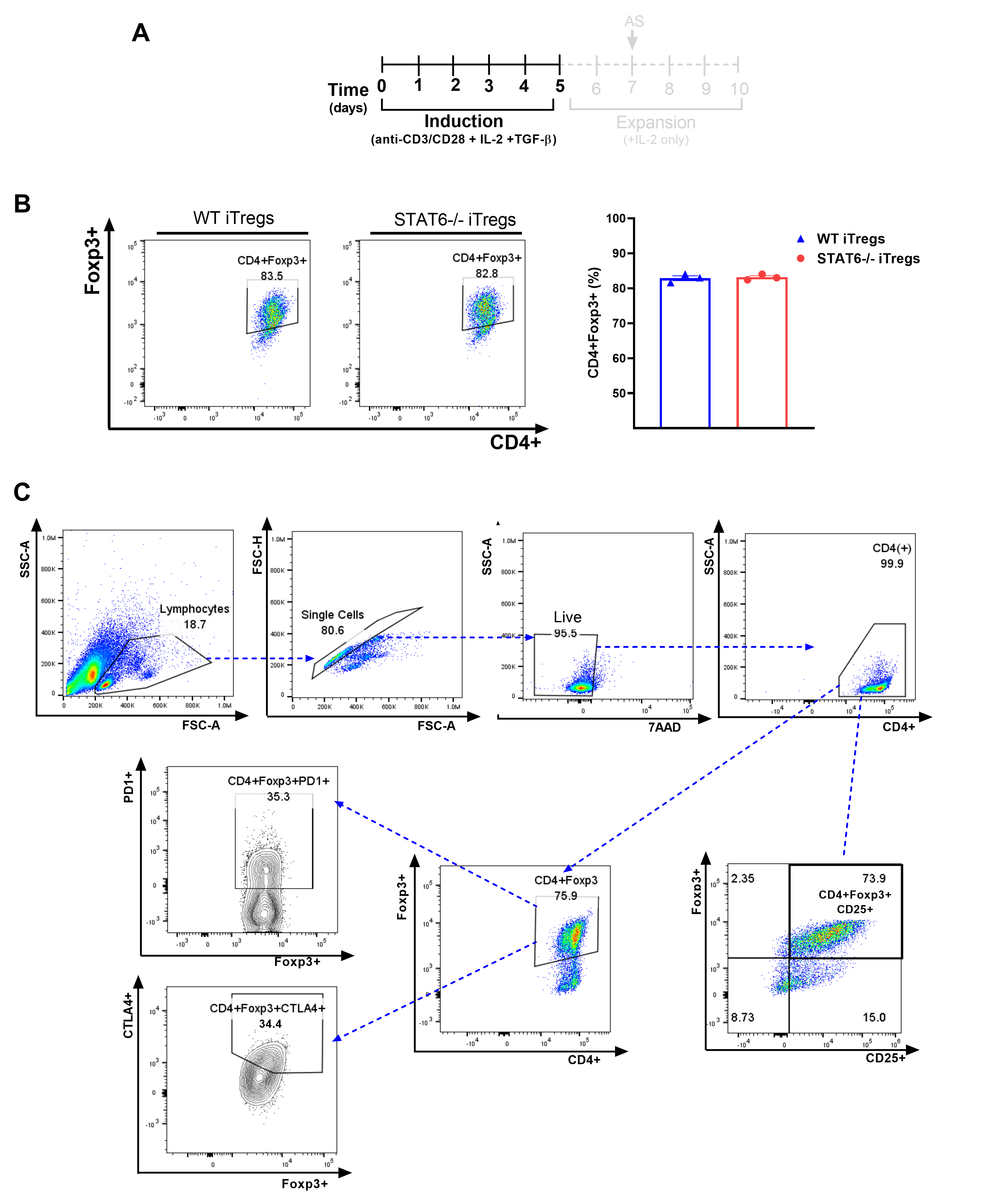

Supplement: Supplementary file 1 — Figure S1 Induction and Analysis of iTregs from CD4⁺ Naïve T Cells. A. Schematic of the 5-day in vitro induction protocol used to generate induced regulatory T cells (iTregs) from CD4⁺ naïve T cells isolated from wild-type (WT) or STAT6⁻/⁻ mice. B. Representative flow cytometry (FCM) plots and corresponding bar graphs showing the frequencies of CD4⁺Foxp3⁺CD25+ iTregs on day 5 post-induction. C. Representative FCM plots illustrating the gating strategy employed for analysis throughout the study. (TIF 1.90 MB) [file 12026_2025_9686_MOESM1_ESM.tif]

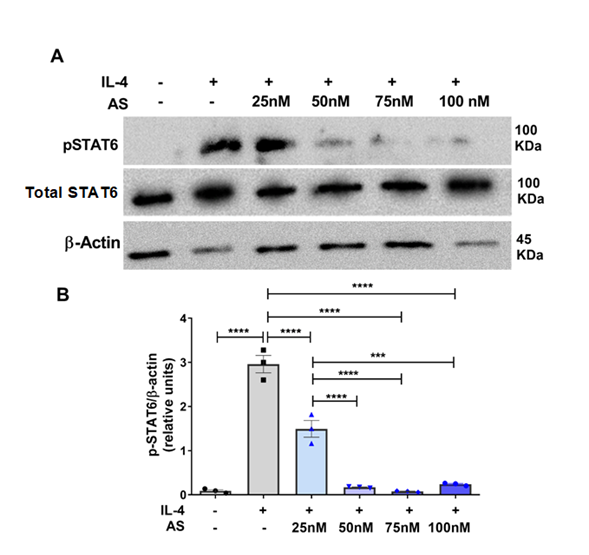

Supplement: Supplementary file 2 — Figure S2 A. Representative Western blots showing the protein levels of phosphorylated STAT6 in WT AS-iTregs. The cells were stimulated with 20 ng/ml recombinant murine IL-4 for 20 min and 24 h with and without AS1517499 (AS). B. Densitometry analysis of pSTAT6 expression under the conditions previously mentioned. Densitometry analyses of Western blots show the results of at least two independent experiments. (***p < 0.001, ****p < 0.0001) (TIF 142 KB) [file 12026_2025_9686_MOESM2_ESM.tif]
